# Curcumin inhibits HCV replication by induction of heme oxygenase-1 and suppression of AKT

**DOI:** 10.3892/ijmm.2012.1096

**Published:** 2012-08-20

**Authors:** MING-HO CHEN, MING-YANG LEE, JING-JING CHUANG, YI-ZHEN LI, SIN-TZU NING, JUNG-CHOU CHEN, YI-WEN LIU

**Affiliations:** 1 Departments of Chinese Medicine and; 2 Hematology and Oncology, Ditmanson Medical Foundation Chia-Yi Christian Hospital, Chiayi;; 3 Department of Medical Laboratory Science and Biotechnology, Chung Hwa University of Medical Technology, Tainan;; 4 Department of Microbiology, Immunology and Biopharmaceuticals, National Chiayi University, Chiayi;; 5 School of Post Baccalaureate Chinese Medicine, Chinese Medical University, Taichung;; 6 The School of Chinese Medicine for Post-Baccalaureate, I-SHOU University, Kaohsiung, Taiwan, R.O.C.

**Keywords:** hepatitis C, curcumin, heme oxygenase-1, AKT, extracellular signal-regulated kinases, nuclear factor-κB

## Abstract

Although hepatitis C virus (HCV) affects approximately 130–170 million people worldwide, no vaccines are available. HCV is an important cause of chronic hepatitis, cirrhosis and hepatocellular carcinoma, leading to the need for liver transplantation. In this study, curcumin, a constituent used in traditional Chinese medicine, has been evaluated for its anti-HCV activity and mechanism, using a human hepatoma cell line containing the HCV genotype 1b subgenomic replicon. Below the concentration of 20% cytotoxicity, curcumin dose-dependently inhibited HCV replication by luciferase reporter gene assay, HCV RNA detection and HCV protein analysis. Under the same conditions, curcumin also dose-dependently induced heme oxygenase-1 with the highest induction at 24 h. Hemin, a heme oxygenase-1 inducer, also inhibited HCV protein expression in a dose-dependent manner. The knockdown of heme oxygenase-1 partially reversed the curcumin-inhibited HCV protein expression. In addition to the heme oxygenase-1 induction, signaling molecule activities of AKT, extracellular signal-regulated kinases (ERK) and nuclear factor-κB (NF-κB) were inhibited by curcumin. Using specific inhibitors of PI3K-AKT, MEK-ERK and NF-κB, the results suggested that only PI3K-AKT inhibition is positively involved in curcumin-inhibited HCV replication. Inhibition of ERK and NF-κB was likely to promote HCV protein expression. In summary, curcumin inhibited HCV replication by heme oxygenase-1 induction and AKT pathway inhibition. Although curcumin also inhibits ERK and NF-κB activities, it slightly increased the HCV protein expression. This result may provide information when curcumin is used as an adjuvant in anti-HCV therapy.

## Introduction

Hepatitis C virus (HCV) affects approximately 130–170 million people worldwide (1), however, no vaccines are available. It is an important cause of chronic hepatitis, cirrhosis, hepatocellular carcinoma (HCC), leading to a need for liver transplantation (2,3). Treatment of chronic HCV is currently based on the combination of pegylated interferon (IFN)-α and the nucleotide analogue ribavirin, which is only effective in approximately 50% of the patients, especially in HCV genotype 1 (4,5). HCV belongs to the *Hepacivirus* genus within the *Flaviviridae* family, and is a positive-stranded RNA virus with a genome of ∼9.6 kb. The HCV genome contains a single open reading frame (ORF) encoding a large polyprotein precursor of 3011 amino acids. The ORF is flanked by 5′ and 3′ untranslated regions. The precursor polyprotein is processed by cellular and viral proteases into 10 proteins: structural (core, E1 and E2), and non-structural proteins (p7, NS2, NS3, NS4A, NS4B, NS5A and NS5B) (3,6). There are six major genotypes in HCV classification (3). The major prevalent type in Southern Taiwan is HCV 1b, which is the most resistant type to interferon therapy (5,7).

Curcumin, derived from eastern traditional medicines, *Curcuma longa*, has been found to have a variety of beneficial properties, such as anti-inflammatory, antioxidant, chemopreventive and chemotherapeutic activities (8,9). Its multiple-target characteristics influence several activities of intracellular molecules, including transcription nuclear factor-κB (NF-κB), pro-inflammatory cyclooxygenase-2 and MAPK inhibitions, as well as heme oxygenase-1 induction (9). In the antivirus bioactivity, certain reports have indicated that curcumin showed anti-viral activity against the human immunodeficiency (10,11), the coxsackie- (12) and the hepatitis B (HBV) viruses (13). In the anti-HCV study, one report showed that curcumin inhibited a lipogenic transcription factor, sterol regulatory element binding protein-1 (SREBP-1)-induced HCV replication via the inhibition of the PI3K-AKT pathway (14).

The catabolism of heme by heme oxygenase (HO) resulted in the production of biliverdin, carbon monoxide and free iron. HO-1, one of the phase II enzymes, is an enzyme in cells with cytoprotective properties against oxidative damage (15) that has been reported to be induced by the Nrf2 transcription factor (16). Curcumin-induced HO-1 expression was first found in human endothelial cells (17), suggesting that a low dose of curcumin induced HO-1 expression, which provided an intrinsic antioxidant ability. Curcumin also induced HO-1 expression in mesangial (18) and liver cells (19–21), as well as in macrophages (22,23). The induction or overexpression of HO-1 has been shown to interfere with the replication of certain viruses, such as the human immunodeficiency virus (24), the HBV (25) and the HCV (26–28).

The properties of the transcription factor NF-κB are extensively exploited in cells (29). In general, NF-κB is of great importance in signal transduction pathways involved in chronic and acute inflammatory diseases, as well as various types of cancer, therefore, it is a good target for cancer prevention (30). Various reports have demonstrated the correlation between curcumin and NF-κB. One of those reports suggests the anti-inflammatory effect of curcumin, which suppresses the ox-LDL-induced MCP-1 expression via the p38 MAPK and NF-κB pathways in rat vascular smooth muscle cells (31). The anti-inflammatory effect of curcumin has been reported to be due to the IκB/NF-κB system in rat and human intestinal epithelial cells, including IEC-6, HT-29 and Caco-2 cells (32). Curcumin has also been found to have anti-metastatic properties via the inhibition of NF-κB in the highly invasive and metastatic MDA-MB-231 breast cancer cell line (33). Another signaling pathway, Raf/MEK/extracellular signal-regulated kinases (ERK), is of crucial importance in the regulation of cell growth, differentiation, survival, as well as the transmission of oncogenic signals (34). This pathway has also been reported to be a target of curcumin. For example, curcumin inhibited connective tissue growth factor gene expression by suppressing ERK signaling in activated hepatic stellate cells (35). Moreover, curcumin inhibited phorbol myristate acetate-induced MCP-1 gene expression by inhibiting ERK and NF-κB activities in U937 cells (36). However, the manner in which curcumin affects the activities of NF-κB and ERK in HCV-infected hepatoma cells has yet to be determine.

Only one study suggesting that curcumin inhibited HCV replication by suppressing the AKT-SREBP-1 pathway is currently available (14). In this study, the correlation between curcumin-inhibited HCV replication, HO-1, AKT, ERK and NF-κB molecules was examined.

## Materials and methods

### Cell culture and reagents

Huh7.5 cells expressing the HCV genotype 1b subgenomic replicon (Con1/SG-Neo(I) hRlucFMDV2aUb) containing *Renilla* luciferase reporter, kindly provided by Apath, were cultured in Dulbecco’s Modified Eagle’s Medium (DMEM) with 10% fetal bovine serum (FBS), 100 U/ml penicillin, 100 mg/ml streptomycin and 0.5 mg/ml G418. The nuclear extraction kit was purchased from Chemicon (Temecula, CA, USA). Curcumin (Acros Organics, Geel, Belgium), LY294002, U0126 and Ro1069920 were purchased from Tocris (Bristol, UK), and dissolved in dimethyl sulfoxide (DMSO), then added into culture medium containing 0.1% DMSO.

### Cell viability assay

Cell viability was determined by colorimetric MTT assay. Cells were cultured on 24-well plates at a density of 1×10^5^ cells/well. After 24 h, the cells were incubated with varying concentrations of curcumin or 0.1% DMSO for another 24 h. MTT was added to medium for 2 h, the medium was discarded and DMSO was then added to dissolve the formazan product. Each well was measured by light absorbance at 490 nm. The result was expressed as a percentage, relative to the 0.1% DMSO-treated control group.

### Luciferase reporter assay

Cells were subcultured at a density of 4×10^5^ cells/well in 1 ml of culture medium in a 12-well plastic dish for 6 h. Curcumin or DMSO was added to the medium for 24 h. The cells were lysed and cell lysates were prepared for a *Renilla* luciferase assay (Promega, Madison, WI, USA) and protein concentration assays, with Bio-Rad protein assay (Bio-Rad, Hercules, CA, USA). The relative luciferase activities were normalized to the same protein concentration.

### Real-time RT-PCR analysis

Total RNA was isolated from Huh7.5 cells expressing the HCV genotype 1b subgenomic replicon. Reverse transcription (RT) was performed on 2 μg of total RNA by 1.5 μM random hexamer and RevertAid™ reverse transcriptase (Fermentas, Glen Burnie, MD, USA). Then, 1/20 volume of reaction mixture was used for quantitative real-time PCR with HCV specific primers: 5′-AGCGTCTAGCCATGGCGT-3′ and 5′-GGTGTACTCACCGGTTCCG-3′, and GAPDH specific primers: 5′-CGGATTTGGTCGTATTGG-3′ and 5′-AGATGGT GATGGGATTTC-3′, as the endogenous control. The quantitative real-time PCR was followed by Maxima™ SYBR-Green qPCR Master Mix (Fermentas). Real-time PCR reactions contained optimal volume of the reverse transcription mixture, 600 nM each forward and reverse primer and 1X SYBR-Green qPCR Master Mix in 25 μl. Reactions were incubated for 40 cycles in an ABI GeneAmp^®^ 7500 Sequence Detection System, with an initial denaturization step at 95°C for 10 min, followed by 40 cycles of 95°C for 15 sec and 63°C for 1 min. PCR product accumulation was monitored at several points during each cycle, by measuring the increase in fluorescence. Gene expression changes were assessed using the comparative Ct method. The relative amounts of mRNA for HCV were optimized by subtracting the Ct values of HCV from the Ct values of GAPDH mRNA (ΔCt). The ΔCt of the control group was then subtracted from the ΔCt of the curcumin-treated groups (ΔΔCt). Data were expressed as relative levels of HCV RNA.

### Western blotting

For western blotting, analytical 10% sodium dodecyl sulfate (SDS)-polyacrylamide slab gel electrophoresis was performed. Tissue extracts were prepared and a 30–60 μg aliquot of protein extracts was analyzed. For immunoblotting, proteins in the SDS-PAGE gels were transferred to a polyvinylidene difluoride membrane using a trans-blot apparatus. Antibodies against HCV NS5A and HCV NA5B (Santa Cruz Biotechnology, Inc., Santa Cruz, CA, USA), HO-1 (Assay Designs, Inc., Ann Arbor, MI, USA), pAKT (308) and pERK (Santa Cruz Biotechnology, Inc.), NF-κB (Cell Signaling Technology, Beverly, MA, USA), Sp1 (Millipore, Darmstadt, Germany), α-tubulin (GeneTex, Inc., Irvine, CA, USA) and β-actin (Sigma-Aldrich, St. Louis, MO, USA) were used as the primary antibodies. Mouse, rabbit or goat IgG antibodies coupled with horseradish peroxidase were used as the secondary antibodies. An enhanced chemiluminescence kit and VL Chemi-Smart 3000 were used for detection, while the quantity of each band was determined using MultiGauge software.

### HO-1 knockdown by siRNA

Cells (3×10^6^) were seeded in 10-cm dishes for 6 h, then negative control small interfering (siRNA) (10 nM) or HO-1 siRNA (10 nM) (Invitrogen) was transfected into cells using the RNAiMAX Transfection Reagent (Invitrogen), according to the manufacturer’s instructions. Subsequent to adding siRNA for 6 h, the medium was changed to fresh condition medium for 18 h. Then the transfected cells were then analyzed by western blotting.

### Statistical analysis

Data were expressed as the mean ± SE. Statistical evaluation was carried out by one-way ANOVA followed by Dunn’s test. All statistics were calculated using SigmaStat version 3.5 (Systat Software). P<0.05 was considered to indicate a statistically significant difference.

## Results

### Cytotoxicity of curcumin in Huh7.5 cells expressing the HCV genotype 1b subgenomic replicon (Huh7.5-HCV cells)

**Figure 1.**
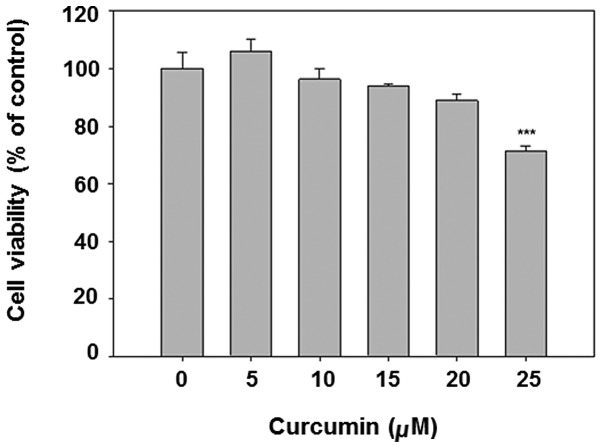
Cytotoxicity of curcumin in Huh7.5-HCV cells is shown. Cells were initially seeded at 1×10^5^ cells/well in 24-well plates, then treated with varying concentrations of curcumin or vehicle (0.1% DMSO), for 24 h. Cell viability was measured by MTT assay. Measurement was obtained from three independent experiments. (^***^P<0.001 compared to vehicle).

Curcumin is known to be an anticancer chemical at high doses. To avoid the obvious cytotocicity in the subsequent experiments, the MTT assay was applied for cytotoxicity analysis. The results show that curcumin dose-dependently decreased cell viability (Fig. 1). The dose <20 μM was selected for subsequent analysis, given that the viability of 25 μM curcumin treatment is <80%.

### Curcumin reduced HCV replication and HCV protein expression

**Figure 2.**
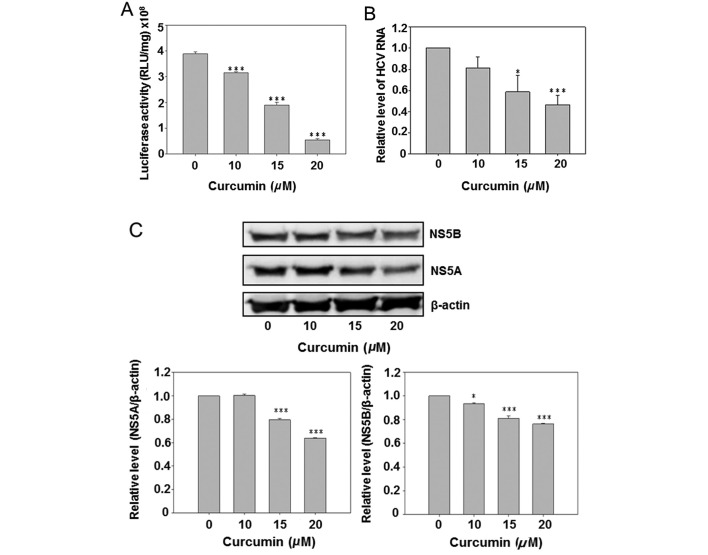
Curcumin dose-dependently inhibits HCV replication. (A) Curcumin inhibits luciferase reporter gene activity in Huh7.5-HCV cells. Cells were subcultured at a density of 4×10^5^ cells/well in 1 ml of culture medium in a 12-well plastic dish for 6 h. Curcumin or DMSO was added to the medium for 24 h. The cells were lysed and cell lysates were prepared for *Renilla* luciferase assay. (B) Curcumin inhibits HCV RNA expression in Huh7.5-HCV cells. Cells were subcultured at a density of 1.5×10^6^ cells in 8 ml of culture medium in a 6-cm plastic dish for 6 h. Curcumin or DMSO was added to the medium for 24 h. Total RNA was isolated and analyzed by real-time RT-PCR. (C) Curcumin inhibits HCV protein expression in Huh7.5-HCV cells. Cells were subcultured at a density of 1.5×10^6^ cells in 8 ml of culture medium in a 10-cm plastic dish for 6 h. Curcumin or DMSO was added to the medium for 24 h. Total protein was isolated and analyzed by western blot analysis. Measurement was performed in triplicate and was repeated three times.

Due to the presence of a luciferase reporter gene in the HCV subgenomic replicon of Con1/SG-Neo(I)hRlucFMDV2aUb, the culture medium luciferase activity was first analyzed subsequent to curcumin treatment. The results show that curcumin dose-dependently inhibited luciferase activity (Fig. 2A). However, the HCV RNA was also detected by real-time PCR. Curcumin also reduced the intracellular HCV RNA expression in a dose-dependent manner. Subsequent to curcumin treatment the HCV-specific protein NS5A and NS5B were detected by western blot analysis, indicating that curcumin dose-dependently inhibited expression of the NS5A and NS5B. The above data suggest that curcumin inhibited HCV replication in hepatoma cells.

### Curcumin induced HO-1 protein expression

**Figure 3.**
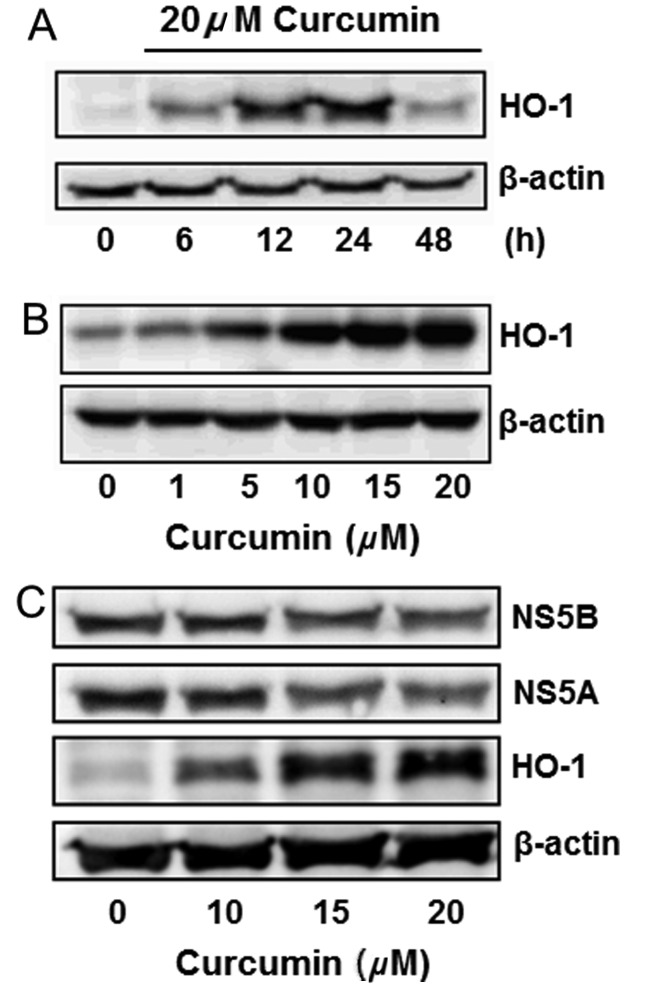
Curcumin induces HO-1 protein expression in Huh7.5-HCV cells. (A) Time course of curcumin-induces HO-1 protein expression is shown. Cells were subcultured at a density of 1.5×10^6^ cells in 8 ml of culture medium in a 10-cm plastic dish for 6 h. Curcumin or DMSO was added to the medium for 6–48 h. Total protein was isolated and analyzed by western blot analysis. (B) Dose-dependent induction of HO-1 by curcumin is shown. Cells were subcultured at a density of 1.5×10^6^ cells in 4 ml of culture medium in a 10-cm plastic dish for 6 h. Curcumin or DMSO was added to the medium for 24 h. Total protein was isolated and analyzed by western blot analysis. (C) Effect of curcumin on the expression of HO-1 and HCV proteins is shown. Cells were subcultured at a density of 1.5×10^6^ cells in 8 ml of culture medium in a 10-cm plastic dish for 6 h. Curcumin or DMSO was added to the medium for 24 h. Total protein was isolated and analyzed by western blot analysis. The experiments were repeated three times.

Curcumin is known to induce HO-1 expression in various cells. This effect was analyzed in Huh7.5-HCV cells. Curcumin slightly induced HO-1 expression in a 6-h treatment, while significantly inducing it in 12 and 24 h. The HO-1 induction declined after treatment for 48 h (Fig. 3A). Curcumin also induced HO-1 expression in a dose-dependent manner (Fig. 3B). The change of NS5A, NS5B and HO-1 protein expressions was simultaneously detected by western blot analysis, indicating that curcumin dose-dependently inhibited the expression of NS5A and NS5B, while increasing the HO-1 expression (Fig. 3C).

### Hemin reduced HCV replication and the HCV protein expression

**Figure 4.**
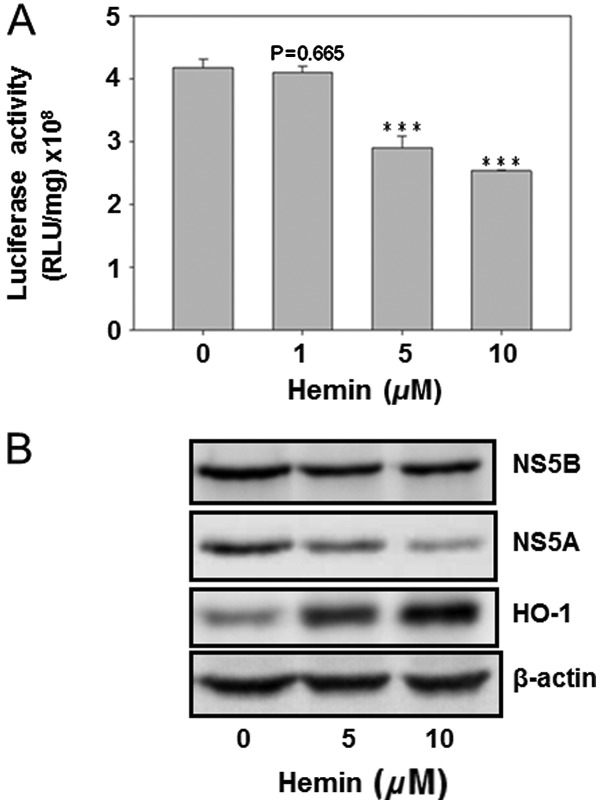
Hemin dose-dependently inhibits HCV replication. (A) Hemin inhibits luciferase reporter gene activity in Huh7.5-HCV cells. Cells were subcultured at a density of 4×10^5^ cells/well in 1 ml of culture medium in a 12-well plastic dish for 6 h. Hemin or DMSO was added to the medium for 24 h. The cells were lysed and cell lysates were prepared for the *Renilla* luciferase assay. (B) Effect of hemin on the expression of HO-1 and HCV proteins is shown. Cells were subcultured at a density of 1.5×10^6^ cells in 8 ml of culture medium in a 10-cm plastic dish for 6 h. Hemin or DMSO was added to the medium for 24 h. Total protein was isolated and analyzed by western blot analysis. The experiments were repeated three times.

The HO-1 inducer hemin was used to analyze its effect on HCV replication as well as on the protein expression of HCV NS5A and NS5B. The result showed that hemin dose-dependently decreased HCV replication (Fig. 4A). Furthermore, curcumin inhibited the protein expression of NS5A and NS5B, while enhancing the HO-1 protein expression. This finding suggested that HO-1 protein inhibited HCV replication in Huh7.5-HCV cells (Fig. 4).

### HO-1 knockdown partially reversed the curcumin-reduced viral protein expression

**Figure 5.**
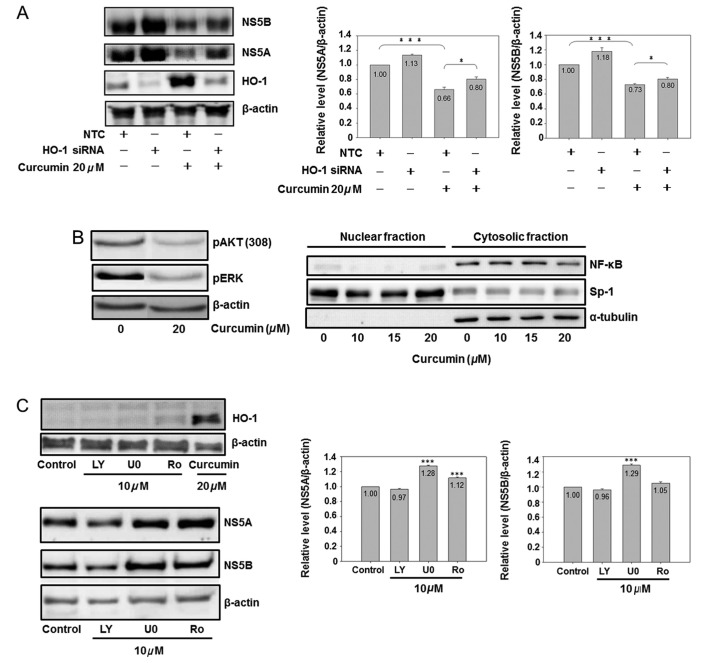
The role of HO-1, AKT, ERK and NF-κB on curcumin-inhibited HCV protein expression is shown. (A) Knockdown of HO-1 partially reversed curcumin-inhibited HCV protein expression. Cells (3×10^6^) were seeded in a 10-cm dish for 6 h, and negative control small interfering (siRNA) (10 nM) or HO-1 siRNA (10 nM) was transfected into cells. Subsequent to a 6-h addition of siRNA, the medium was changed to fresh condition medium for 18 h, and the transfected cells were analyzed by western blotting (*P<0.05 and ^***^P<0.001, in 2 groups, respectively). (B) Curcumin inhibited AKT, ERK and NF-κB. Cells were subcultured at a density of 1.5×10^6^ cells in 8 ml of culture medium in a 10-cm plastic dish for 6 h. Curcumin or DMSO was added to the medium for 24 h. Total cell lysates (up) or cytosol-nuclear fraction (down) were isolated by western blot analysis. Sp1 is a dominant nuclear protein and α-tubulin is a cytosolic protein. (C) Effect of AKT, ERK and NF-κB inhibitors on the HCV protein expression is shown. Cells were subcultured at a density of 1.5×10^6^ cells in 8 ml of culture medium in a 10-cm plastic dish for 6 h. Chemical (LY, LY294002; U0, U0126; Ro, Ro1069920) or DMSO was added to the medium for 24 h. Total cell lysates were isolated for western blot analysis. (^***^P<0.001 compared to control). The experiments were repeated three times.

In order to prove the direct relationship between curcumin-induced HO-1 and curcumin-inhibited HCV replication, the HO-1 specific siRNA was used for analysis. HO-1 siRNA significantly inhibited basal and curcumin-induced HO-1 expression (Fig. 5A). HO-1 knockdown slightly increased the NS5A and NS5B protein expressions in the basal condition. At the same time, it partially but significantly reversed the curcumin-inhibited the expression of NS5A and NS5B, suggesting that curcumin-induced HO-1 was involved in curcumin-inhibited HCV replication, while having additional mechanisms regarding the anti-HCV effect of curcumin.

### Effect of the PI3K-AKT, MEK-ERK and NF-κB pathways on curcumin-inhibited HCV replication

Fig. 5A shows that HO-1 is partially involved in curcumin-inhibited HCV replication. Additional signaling pathways affected by curcumin were analyzed, demonstrating that curcumin inhibited the protein phosphorylation of ERK and AKT, as well as the cytoplasmic protein expression of NF-κB (Fig. 5B). Therefore, the specific inhibitors of PI3K-AKT (LY294002), MEK-ERK (U0126) and NF-κB (Ro 106-9920) were used to identify the role of AKT, ERK and NF-κB in the HCV protein expression. Fig. 5C shows that curcumin was the only chemical to induce the HO-1 expression. Of the three inhibitors, only PI3K-AKT LY294002 slightly inhibited the HCV protein expression, while MEK-ERK U0126 and NF-κB inhibitors Ro 1069920 had a slight effect on increasing the HCV protein expression, suggesting that curcumin-inhibited HCV replication was also partially mediated via PI3K-AKT inhibition.

## Discussion

Curcumin is a common chemical ingredient of curry. It has, however, been studied in clinical trials regarding its applicability in treating patients suffering from pancreatic and colon cancer, as well as multiple myeloma (37). In Taiwan, several doctors of traditional Chinese medicine consider curcumin to be beneficial for patients suffering from hepatitis. The results of this study demonstrate that curcumin inhibits HCV replication in cellular analysis, and its mechanism partially occurs through HO-1 induction and PI3K-AKT inhibition.

HO-1, a curcumin-induced gene, is thought to be a potential therapeutic protein for the re-establishment of homeostasis in several pathologic conditions (38) and is also involved in inhibiting HCV replication (28). The HO-1 products biliverdin and iron contribute to certain anti-HCV mechanisms of HO-1 (26,39,40). In this study, HO-1 knockdown partially reversed curcumin-inhibited HCV replication, supporting the evidence for the anti-HCV effect of HO-1. Since HO-1 is induced by ROS or certain electrophiles, ROS has also been reported to inhibit HCV replication (41,42). Arsenic trioxide-inhibited HCV replication is also suggested to be mediated through the induction of oxidative stress (43). HO-1, an oxidative stress-induced gene, may be involved in the ROS-inhibited HCV replication.

As a downstream kinase of PI3K, AKT is an important molecule in regulating a wide range of signaling pathways (44). In HCV-infected cells, the PI3K-AKT signaling pathway is involved in certain pathological mechanisms. For example, the activities of PI3K, AKT and their downstream target mTOR are increased in the HCV-replicating cells (45). HCV NS5A binds to PI3K, while enhancing the phosphotransferase activity of the catalytic domain (46). The HCV-activated PI3K-AKT contributes to cell survival enhancement. In addition to cell survival, AKT leads to the protein accumulation of SREBP-1, an important transcription factor regulating genes involved in fatty acid and cholesterol synthesis (47). HCV NS4B has been found to enhance the protein expression levels of SREBPs and fatty acid synthase through PI3K activity, subsequently inducing a lipid accumulation in hepatoma cells (48). Therefore, inhibition of the PI3K-SREBP signaling pathway should decrease the HCV-induced HCC development and the cellular fatty acid level. Curcumin has been reported to inhibit HCV replication via suppression of the AKT-SREBP-1 pathway (14). In the present study, data also demonstrated that curcumin-inhibited PI3K-AKT was slightly involved in the anti-HCV activity of curcumin.

Activation of the MEK-ERK signal cascade enhances the replication of viruses, such as the human immunodeficiency (49), the influenza (50), the corona- (51) and the herpes simplex viruses (52). By contrast, in the case of HBV, activation of MEK-ERK signaling led to the inhibition of HBV replication (53). In the HCV study, interleukin-1 has been reported to have the potential to effectively inhibit HCV replication and protein expression by activating the ERK signaling pathway (54). HCV IRES-dependent protein synthesis was enhanced by MEK-ERK inhibitor PD98059 (55). Another report also suggests that inhibition of MEK-ERK signaling leads to the upregulation of HCV replication and protein production (56). Consistent with the results of the present study, those findings confirm that the curcumin-inhibited MEK-ERK signaling pathway contributes to the increase of HCV replication.

NF-κB, one of the major signaling transduction molecules activated in response to oxidative stress, is able to modulate the transcription of a large number of downstream genes. The HCV core protein has been shown to activate NF-κB, inducing resistance to TNF-α-induced apoptosis in hepatoma cells (57). HCV NS2 activates the IL-8 gene expression by activating the NF-κB pathway in HepG2 cells (58). In the infectious JFH1 model, HCV is suggested to enhance hepatic fibrosis progression through the induction of TGF-β1, mediated by a ROS-induced and NF-κB-dependent pathway (59). These evidences indicate that the activation of NF-κB by HCV induces hepatic disease progression. In this study, the NF-κB expression is abundant in the cytoplasm of Huh7.5 cells, expressing the HCV genotype 1b subgenomic replicon (Fig. 5B). The absence of NF-κB nuclear translocation indicates that NF-κB is not likely to participate in the mechanism of hepatocarcinogenesis in this cell line. The absense of complete HCV core and HCV NS2 sequences in the subgenomic replicon used in this study, is likely to be the reason for the absence of NF-κB nuclear translocation. Therefore, it is likely to contribute to the inability of the NF-κB inhibitor to suppress the HCV protein expression in this cell line. In fact, the genomic variation of HCV core protein generates a distinct functional regulation of NF-κB, which may inhibit or activate NF-κB activity (60).

In certain reports, the inhibition of NF-κB shows anti-HCV activity: for example, the *Acacia confusa* (61) and San-Huang-Xie-Xin-Tang extracts (62) suppress HCV replication associated with NF-κB inhibition. In the present study, curcumin-inhibited NF-κB does not have any benefit in anti-HCV activity. Thus, the presence or absence of the inhibition of NF-κB in anti-HCV therapy is likely to depend on the activation status of NF-κB, although additional investigations are required on the subject.

In conclusion, this study proved that curcumin inhibits HCV replication through the induction of the HO-1 expression and the inhibition of the PI3K-AKT signaling pathway. However, the curcumin-inhibited MEK-ERK mechanism contributes negatively to its anti-HCV activity.
